# Phenotypic and Molecular Characterization of an Enterobacter ludwigii Clinical Isolate Carrying a Plasmid-Mediated *bla*_IMI-6_ Gene

**DOI:** 10.1128/spectrum.04620-22

**Published:** 2023-04-19

**Authors:** T. Blanco-Martín, J. Guzmán-Puche, C. Riazzo, M. Gasca-Santiyán, M. Hernández-García, R. Cantón, J. Torre-Cisneros, C. Herrera, L. Martínez-Martínez

**Affiliations:** a Microbiology Unit, Reina Sofía University Hospital, Cordoba, Spain; b Maimonides Biomedical Research Institute of Cordoba (IMIBIC), Cordoba, Spain; c CIBER de Enfermedades Infecciosas (CIBERINFEC), Instituto de Salud Carlos III, Madrid, Spain; d Microbiology Unit, Ramón y Cajal University Hospital and Ramón y Cajal Institute for Health Reasearch (IRICYS), Madrid, Spain; e Infectious Diseases Unit, Reina Sofía University Hospital, Cordoba, Spain; f Medical and Surgical Sciences Department, University of Cordoba, Cordoba, Spain; g Haematology Department, Reina Sofía University Hospital, Cordoba, Spain; h Agricultural Chemistry, Soil Science and Microbiology Department, University of Cordoba, Cordoba, Spain; JMI Laboratories

**Keywords:** *Enterobacter ludwigii*, IMI-6, carbapenemase

## Abstract

We report a plasmid-encoded IMI-6 carbapenemase in a clinical isolate of Enterobacter ludwigii from Spain. The isolate belongs to ST641 and was susceptible to expanded-spectrum cephalosporins and resistant to carbapenems. The modified carbapenem inactivation method (mCIM) test was positive, but β-Carba was negative. Whole-genome sequencing identified the *bla*_IMI-6_ gene located in a conjugative IncFIIY plasmid and associated with the LysR-like regulator *imiR*. Both genes were bracketed by an IS*EclI*-like insertion sequence and a putatively defective IS*Ec36* insertion sequence.

**IMPORTANCE** IMI carbapenemases confer an unusual resistance pattern of susceptibility to broad-spectrum cephalosporins and piperacillin-tazobactam but decreased susceptibility to carbapenems, which may make them difficult to detect in routine practice. Commercially available molecular methods for the detection of carbapenemases in clinical laboratories do not usually include *bla*_IMI_ genes, which could contribute to the hidden dissemination of bacteria producing these enzymes. Techniques should be implemented to detect minor carbapenemases that are not very frequent in our environment and control their dissemination.

## OBSERVATION

The increasing incidence of carbapenem-resistant *Enterobacterales* (CRE) is a worldwide public health problem (1). IMI enzymes are class A carbapenemases which have been infrequently detected in Asia, Europe, and America (2–17), most often in Enterobacter spp. In most cases, genes coding for these enzymes are chromosomally located (2), but some genes, such as *bla*_IMI-2_, *bla*_IMI-3_, *bla*_IMI-5_, and *bla*_IMI-6_, have also been described in conjugative plasmids (3–5). Many commercial diagnostic tests do not detect IMI enzymes, which is likely leading to an underestimation of their frequency among clinically relevant Gram-negative pathogens. We report the genetic characterization of a plasmid-mediated IMI-6 carbapenemase in a clinical isolate of Enterobacter ludwigii from Spain.

In October 2021, a carbapenem-resistant organism (HURS_212964) was isolated from a rectal swab culture during a surveillance study for CRE in a 77-year-old patient with a Burkitt lymphoma. The patient was admitted to the hematology unit of our hospital for a cycle of programmed polychemotherapy according to the Burkimab scheme (18), which he received without any infections or other complications. He was discharged after 5 days of hospitalization. The isolate was initially identified by matrix-assisted laser desorption ionization–time of flight (MALDI-TOF) (MALDI Biotyper A system; Bruker Daltonics, Madrid, Spain) as Enterobacter cloacae complex.

Antimicrobial susceptibility testing was determined by broth microdilution using Sensititre DKMNG and EUMDROXF panels (Thermo Fisher Scientific, Waltham, MA, USA). MIC values were interpreted according to EUCAST guidelines (19). The HURS_212964 isolate was susceptible to ceftazidime (MIC, ≤0.5 mg/L), cefotaxime (MIC, ≤0.5 mg/L), cefepime (MIC, ≤1 mg/L), piperacillin-tazobactam (MIC, 4 mg/L), ceftazidime-avibactam (MIC, ≤0.25/4 mg/L), ceftolozane-tazobactam (MIC, 1/4 mg/L), imipenem-relebactam (MIC, 1/4 mg/L), meropenem-vaborbactam (MIC, ≤0.06/8 mg/L), gentamicin (MIC, ≤0.5 mg/L), tobramycin (MIC, ≤1 mg/L), amikacin (MIC, ≤4 mg/L), ciprofloxacin (MIC, ≤0.06 mg/L), colistin (MIC, 0.5 mg/L), and trimethoprim-sulfamethoxazole (MIC, ≤1/19 mg/L), susceptible to aztreonam (MIC, 4 mg/L) and meropenem (MIC, 8 mg/L) when exposure is increased, and resistant to both imipenem (MIC, >8 mg/L) and ertapenem (MIC, >2 mg/L).

Discs (Oxoid, Thermo Fisher Scientific, Madrid, Spain) of cefotaxime (30 μg), ceftazidime (30 μg), and cefepime (30 μg) alone or combined with clavulanic acid (10 μg) on both Mueller-Hinton (MH) agar and MH agar supplemented with 200 mg/L of cloxacillin (bioMérieux S.A., Marcy-l’Étoile, France) did not detect the presence of either an extended-spectrum beta-lactamase (ESBL) or an overexpressed AmpC. Discs of temocillin (30 μg) and meropenem alone (10 μg) or combined with boronic acid, dipicolinic acid, or cloxacillin (Rosco Diagnostica A/S, Taastrup, Denmark) showed synergism of meropenem with boronic acid, suggesting the presence of a class A carbapenemase.

Detection of carbapenemase activity by the modified carbapenem inactivation method (mCIM) was positive (meropenem inhibition zone, 6 mm), but the β-Carba test (Bio-Rad, Madrid, Spain), the NG-Test-CARBA-5 immunochromatography test (Biotech, Madrid, Spain), and a commercial PCR assay (GenXpert; Cepheid, Barcelona, Spain) were all negative.

Whole-genome sequencing (WGS) was performed using Illumina technology. DNA paired-end libraries were generated using the Nextera XT DNA sample preparation kit (Illumina Inc., San Diego, CA, USA). Libraries were sequenced using the Illumina NextSeq500 sequencer system with 2- by 150-bp paired-end reads. Comparison of Enterobacter genomes using average nucleotide identity showed that isolate HURS_212964 belonged to the Enterobacter ludwigii species with a score of 98.9%. The isolate belonged to the sequence type ST641 (6). The presence of the *bla*_IMI-6_ gene in association with the regulator *imiR* was detected on the only identified plasmid belonging to the IncFIIY group. Both genes were found to be flanked by the IS*3*-family insertion sequences IS*Ec36*-like and IS*Ecl1*-like located downstream and upstream of *bla*_IMI-6_ and *imi-6R*, respectively. The IS*Ec36* sequence presented a point mutation leading to a premature stop codon, and the IS*Ecl1* transposase showed a deletion of two amino acids in the OrfB (His293 and Ser294). Sequenced reads were mapped against the previously described *bla*_IMI-6_-carrying plasmid pIMI-6 from the Enterobacter cloacae isolate N14-0444 (accession number KX78618) leading to a ca.-160-kb plasmid named pHURS_212964 that shares high nucleotide sequence identity (>99%) with pIMI-6 and pRJ46C also carrying the *bla*_IMI-6_ gene identified from a Raoultella ornithinolytica isolate (accession number KT225520) (3). Shared sequences included the *umuC-umuD* genes responsible for mutagenesis regulation, *parA-parB* genes for partition, *traM* to *traX* genes for conjugative transfer, and a type VI secretion system (T6SS) region, which is involved in bacterial virulence (see Fig. S1 in the supplemental material). WGS also showed the presence of the chromosomal *bla*_ACT-12_ gene with two amino acid substitutions (Ala214Ser and Gln217Pro).

Plasmid transferability of *bla*_IMI-6_ was assessed by conjugation experiments using Escherichia coli J53 Rif-R as the recipient strain. Transconjugants were selected on MH agar plates containing 100 μg/mL of rifampicin and 100 μg/mL of ampicillin. PCR amplification followed by Sanger sequencing was used to confirm plasmid transfer. E. coli transconjugants carrying *bla*_IMI-6_ confirmed the carbapenem resistance pattern, thus demonstrating that the *bla*_IMI-6_ gene was responsible for carbapenem resistance in the parental Enterobacter ludwigii isolate (Table 1).

**TABLE 1:** Minimal inhibitory concentration (mg/L) of antimicrobial agents against Enterobacter ludwigii HURS_212964, Escherichia coli J53 transconjugant producing IMI-6, and Escherichia coli J53

Antibiotic	Enterobacter ludwigii IMI-6	Escherichia coli J53 IMI-6	Escherichia coli J53
Piperacillin/tazobactam	4/4	2/4	≤1/4
Cefotaxime	≤0.5	≤0.5	≤0.5
Ceftazidime	≤0.5	≤0.5	≤0.5
Ceftazidime/avibactam	≤0.25/4	≤0.25/4	≤0.25/4
Ceftolozane/tazobactam	1/4	1/4	≤0.25/4
Aztreonam	4	4	≤0.5
Ertapenem	>2	>2	≤0.12
Imipenem	>8	>8	≤0.5
Imipenem/relebactam	1/4	0.12/4	≤0.06/4
Meropenem	8	4	≤0.12
Meropenem/vaborbactam	≤0.06/8	≤0.06/8	≤0.06/8
Gentamicin	≤0.5	≤0.5	≤0.5
Tobramycin	≤1	≤1	≤1
Amikacin	≤4	≤4	≤4
Ciprofloxacin	≤0.06	≤0.06	≤0.06
Colistin	0.5	0.5	≤0.25
Trimethoprim/sulfamethoxazole	≤1/19	≤1/19	≤1/19

Although IMI-1 was initially reported in two clinical isolates of E. cloacae cultured in 1984 (2), IMI enzymes are still infrequently described (2–17). These enzymes have been mostly identified in Enterobacter spp. and have also been reported in Raoultella ornithinolytica, Klebsiella variicola, and Escherichia coli (3, 7, 8). There are few reports about IMI carbapenemases in Spain: IMI-2 was first described in an E. coli isolate in 2012 and subsequently reported in 2017 in an Enterobacter cloacae isolate from a river ecosystem (9). In 2018, IMI-1 was also detected in an Enterobacter cloacae isolate from Andalusia (10).

To date, there are 24 assigned variants of IMI enzymes in the GenBank database, which are encoded by chromosomal or plasmid genes (2–17). In our isolate, the *bla*_IMI-6_ gene was identified on a conjugative IncFIIY plasmid; this group of plasmids has also been identified as carriers of *bla*_IMI-2_ and *bla*_IMI-5_ (3, 4). As previously described for all *bla*_IMI-like_ genes, *bla*_IMI-6_ was associated with *imiR*, a LysR-family transcriptional regulator located upstream of the gene (4, 7, 8, 11). These genes were flanked by insertion sequences which may have played a relevant role in the mobilization of these resistance genes.

IMI carbapenemases confer the unusual resistance pattern of susceptibility to broad-spectrum cephalosporins and piperacillin-tazobactam but decreased susceptibility to carbapenems, which may make them difficult to detect in routine practice. IMI-6 was not detected by the β-Carba test, and previous studies also indicate that IMI-6 enzymes are not detected by the Carba-NP test (3). In addition, commercially available molecular methods for the detection of carbapenemases in clinical laboratories do not usually include *bla*_IMI_ genes, which could contribute to the hidden dissemination of bacteria producing these enzymes.

Techniques should be implemented to detect minor carbapenemases that are not very frequent in our environment and control their dissemination.

### Accession number(s).

Sequence data have been deposited in NCBI under accession number JAPHQK000000000.
