# Supplementary figures and images for: Phenotypic and Molecular Characterization of an Enterobacter ludwigii Clinical Isolate Carrying a Plasmid-Mediated blaIMI-6 Gene
Source: Microbiol Spectr. 2023 Apr 19;11(3):e04620-22. doi: 10.1128/spectrum.04620-22 (PMC10269617; doi:10.1128/spectrum.04620-22)

Figure 1. Schematic diagram of pHURS\_212964.

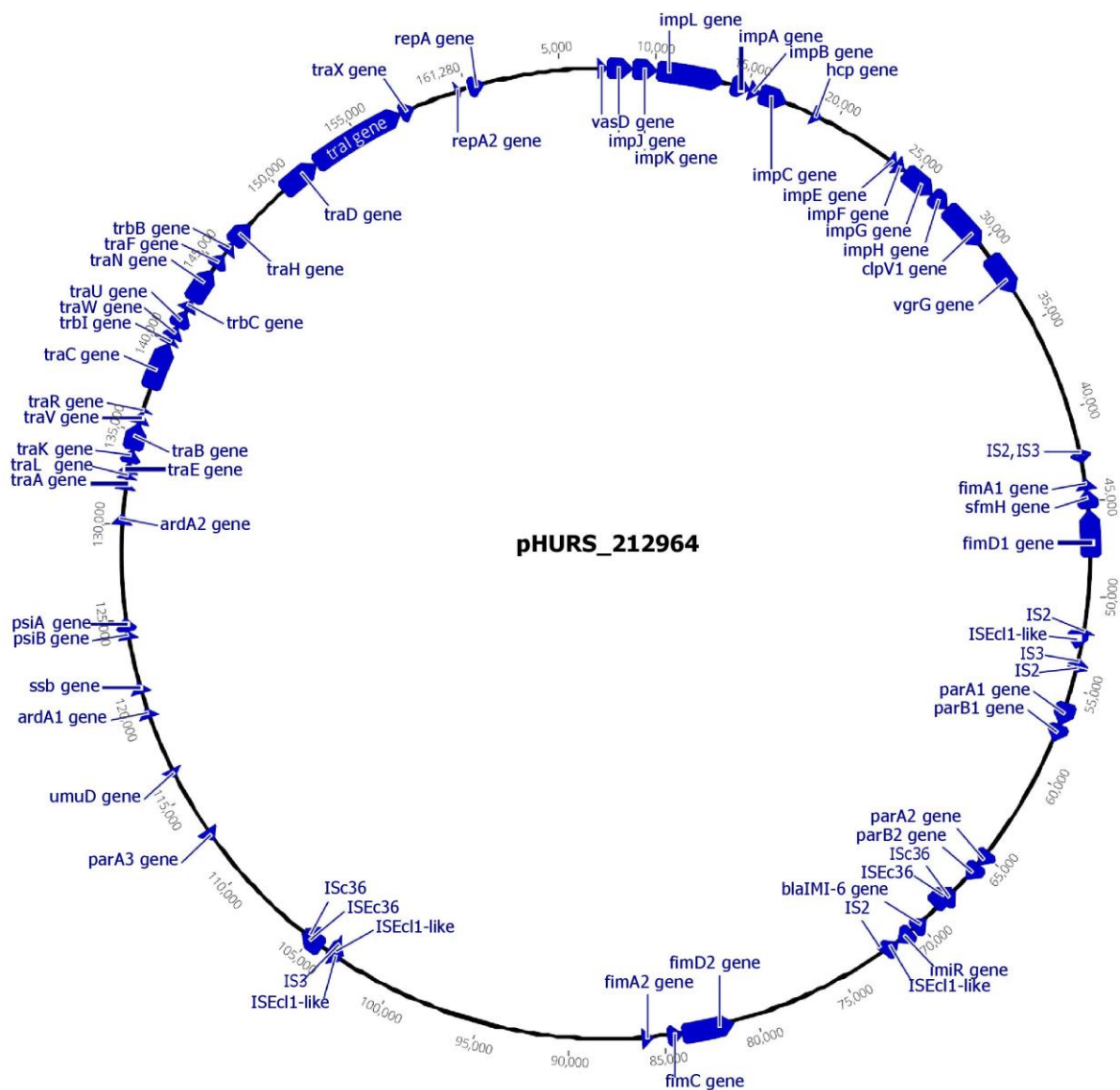

Supplement: Supplemental file 1 — Fig. S1. Download spectrum.04620-22-s0001.pdf, PDF file, 0.2 MB [file spectrum.04620-22-s0001.pdf]
